# How to Use Global Positioning Systems (GPS) Data to Monitor Training Load in the “Real World” of Elite Soccer

**DOI:** 10.3389/fphys.2020.00944

**Published:** 2020-08-20

**Authors:** Guillaume Ravé, Urs Granacher, Daniel Boullosa, Anthony C. Hackney, Hassane Zouhal

**Affiliations:** ^1^Toulouse Football Club, Toulouse, France; ^2^Division of Training and Movement Sciences, University of Potsdam, Potsdam, Germany; ^3^INISA, Federal University of Mato Grosso do Sul, Campo Grande, Brazil; ^4^Department of Exercise & Sport Science; Department of Nutrition, University of North Carolina, Chapel Hill, NC, United States; ^5^Univ Rennes, M2S (Laboratoire Mouvement, Sport, Santé), Rennes, France

**Keywords:** acute chronic workload ratio, injury risk, physical performance, monitoring, external training load

## Introduction

Modern physical training in elite sport is characterized by the systematic and continuous assessment of data on competitive and training performances (Clemente et al., 2019a). In team sports, Global Positioning Systems (GPS) technology is probably the most used monitoring tool to record workloads during training and competitions (Akenhead and Nassis, 2016).

In soccer, the training load (TL) has previously been defined as the input variable that is manipulated to elicit the desired training response (Impellizzeri et al., 2019) and it can be differentiated into external and internal loads (Jaspers et al., 2017). While external TL refers to the overall activities of a player, internal TL encompasses the psycho-physiological stress imposed on the player's body (Jaspers et al., 2017). Both internal and external TLs represent the cumulative exposure of each player to training and competitions (Jaspers et al., 2018). TL can be assessed by means of internal and external measures (Impellizzeri et al., 2019). For internal measures, heart rate or rating of perceived exertion have traditionally been applied (Owen et al., 2015). For external measures, GPS data have proven to be a valid and reliable means (Nikolaidis et al., 2018). GPS measures time motion parameters represented by the distance covered and the number of efforts at different running velocities (e.g., up to 25.2 km/h), as well as bouts of acceleration and deceleration throughout an activity (e.g., up to 3 m/s^2^ or −3 m/s^2^, respectively) at different intensities (Akenhead and Nassis, 2016) over a few meters which are too short to reach high speed running (Varley et al., 2017).

However, it must be emphasized that the concept of GPS metrics based on thresholds have not yet reached consensus in the scientific literature (Rago et al., 2019b). Nevertheless, GPS data are frequently recorded to monitor external TL during game or training situations for each soccer player individually as well as for the whole team (Buchheit and Simpson, 2017). Indeed, evidence suggest that the external TL management is key to maintain physical fitness (e.g., VO_2_max; Clemente et al., 2019b) and match physical performances over time (Lee and Mukherjee, 2019). Thus, the total distance covered correlated negatively with the percentage of changes in mean HR during submaximal aerobic tests (Rago et al., 2019c). Moreover, the time spent in the maximal aerobic speed zone highly correlates with changes in aerobic fitness (Fitzpatrick et al., 2018). Game physical performance is random as it depends on situational factors (e.g., score, match location, opponent; Lago-Peñas, 2012; Redwood-Brown et al., 2018) and players' pitch position (Barnes et al., 2014; Ingebrigtsen et al., 2015; Akenhead et al., 2016). Thereby, monitoring of high-speed running (Sæterbakken et al., 2019) and the capacity to sustain a high number of accelerations and decelerations (Russell et al., 2016) is highly recommended during training and competition (Pettersen et al., 2018).

Beyond physical performance, external TL management is associated with sustaining musculo-tendinous injuries (Colby et al., 2017; Malone et al., 2018a). The acute to chronic workload ratio (ACWR) concept recently introduced by Gabbett (2016), has widely been used in sport science and practice. The ACWR is a mathematical calculation which consists of dividing the TL of the current week (acute TL) by the rolling average TL or exponentially weighted moving averages of the previous 4 weeks (chronic TL; Gabbett, 2016; Williams et al., 2017). Avoiding TL peaks and a progression in the increase of TL is therefore recommended to reduce the injury risk associated to TL (Gabbett, 2020; Griffin et al., 2020; Maupin et al., 2020). Along these lines, a “danger zone” for an increased injury risk has been suggested to exist when the ACWR is between 0.8 and 1.5 (Gabbett, 2016). This means that when the acute load is <0.8 times or is >1.5 times the chronic training load, the injury risk increases during the subsequent week (Gabbett, 2016). Under this situation, it seems that the injury risk can be modified by high levels of aerobic fitness, greater lower body strength, a reduced history of injuries, and a younger age of the soccer players (Malone et al., 2018a; Gabbett, 2020).

In professional soccer, low chronic external TL associated with rapid and significant increases in the acute workload, increases the risk of non-contact injury (Duhig et al., 2016; Bowen et al., 2017, 2019; Colby et al., 2017; Jaspers et al., 2018; Malone et al., 2018a). Of note, the association between the ACWR and injury risk cannot be interpreted as a manner to predict injuries (Griffin et al., 2020). There is also a critical claim against its internal validity (Impellizzeri et al., 2020). The traditional (“coupled”) ACWR calculation includes acute TL in the numerator and the chronic TL in the denominator of the equation (Gabbett, 2016). This can contribute to spurious correlations (Lolli et al., 2019; Windt and Gabbett, 2019). Meanwhile, the ACWR can help practitioners to receive a large picture of the player's TL for not exposing the player to TL errors (Drew and Finch, 2016). Even though critical reports exist on the ACWR (Wang et al., 2020), the International Olympic Committee has recommended using the ACWR to monitor injury and to provide athletes' thresholds to minimize injury occurrence throughout training programs (Soligard et al., 2016).

In modern training approaches (e.g., tactical periodization; Delgado-Bordonau and Mendez-Villanueva, 2012), training sessions combine technical, tactical, mental, and physical aspects on specific soccer drills (Dellal et al., 2012). Furthermore, the physical aspect of soccer drills, manipulation of rules, number of touches, number of players, duration of exercise, coaching encouragement, and pitch area are different parameters that impact their physical demands (Sarmento et al., 2018). On the other hand, during the season, the number and type of injuries, the status of the player (starter or not starter), having an illness or the wellbeing state are different between players (Anderson et al., 2016; Malone et al., 2018b). Thus, external TL management is a dynamic, complex and challenging task for sport scientists and technical staff to combine scientific recommendations inside the complexity of the training process (Bourdon et al., 2017).

Recently, Malone et al. (2017, 2019) provided methodological recommendations on how to collect, interpret, and report GPS data in team sports and thus to avoid some past methodological issues with its use. Nonetheless, an integration of the practical approaches combining scientific knowledge and coaches' expertise from the “field” appears to be lacking. That is, studies have made recommendations according to a specific topic such as external TL or injury prevention (Jaspers et al., 2018; Bowen et al., 2019), physical development (Clemente et al., 2019b), or game demands (Barnes et al., 2014). However, on the “field,” practitioners need to examine recommendations from a multitude of studies to manage their TL prescription and monitoring. Thus, the purpose of this opinion paper is to propose a practical approach for soccer coaches on how to use GPS data for training load monitoring on a team and individual level. We suggest that the planning of external TL should be realized on a monthly, weekly, and daily level in order to reach collective performance. Inside this collective plan, it is important to expose each player to individual external TL to enhance physical performance and lower the risk of sustaining injuries.

## Relevant Parameters From Global Positioning System Data

The selection of reliable and relevant GPS parameters (Table 1) depends on the purported use. GPS parameters are useful to monitor the external TL (e.g., plan external TL, calculate the ACWR), both individually and collectively, to create specific training sessions and to analyze their level of specificity (Dellal et al., 2012). Based on scientific evidence, the total distance covered, the distance covered at high-speed running (HSR) measured between 19.8 and 24.8 km/h, the distance covered at sprint running (SPR) measured over 25.2 km/h, the specific maximal speed (e.g., to record in the game), the number of accelerations (≥3 m/s^2^), and the number of decelerations (≤ −3 m/s^2^) seem to be relevant GPS parameters to monitor the external TL in professional soccer (Akenhead et al., 2016; Varley et al., 2017; Malone et al., 2019). All these GPS parameters represent the volume of training sessions and games (Figueiredo et al., 2018). Total distance covered, high speed running, or sprint running related to the time (expressed in “m/min”) reflect the intensity and are used to design specific training sessions [e.g., rehabilitation sessions (Taberner et al., 2019)] and to delineate their specificity (e.g., physical activity profile similar to the game; Figueiredo et al., 2018; Whitehead et al., 2018). In addition, the distance of high-speed running efforts (i.e., sprints, accelerations, and decelerations) characterize the specifics of the game or the training session (Martín-García et al., 2018). For these GPS parameters, the average, the minimum, and the maximum values should be considered for analyses (Rago et al., 2019b).

**Table 1:** Relevant GPS parameters to monitor training load, create specific training programs, and analyze the specificity of the training session.

**GPS parameters**	**Abbreviations**
Total distance covered	Total Distance
Distance covered at high speed running *(19.8 to 25.2 km/h)*	HSR
Distance covered at sprint running *(≥ 25.2 km/h)*	SPR
Number of accelerating efforts *(≥ 3 m/s^2^)*	Acc
Number of decelerating efforts *(≤ −3 m/s^2^)*	Dec
Maximal speed	Max speed
Individualized moderate speed running *(between 80 and 99.9% of maximal aerobic speed)*	MSR
Individualized high-speed running *(between 100% maximal aerobic speed and 29% anaerobic speed reserve)*	HSR
Individualized sprint running *(between ≥30% anaerobic reserve speed and 100% of the maximal sprint test)*	SPR
Moderate acceleration *(between 50 and 75% of maximal acceleration)*	MO Acc
High acceleration *(≥75% of maximal acceleration)*	HI Acc
Total distance covered divided by time (*M/min)*	–
Distance covered at sprint running divided by time *(m/min)*	–
Distance of one acceleration *(m)*	–
Distance of one deceleration (*m)*	–
Distance of one high speed running (*19.8–25.2 km/h)*	–
Distance of one sprint running (*≥25.2 km/h)*	–

*HSR, individualized high-speed running; LSA, individualized low speed activities; MSR, individualized moderate speed running; SPR, individualized sprint running*.

The speed or acceleration threshold (e.g., high speed running between 19.8 and 24.8 km/h) has been arbitrarily defined and is equal for all players (Rago et al., 2019b). Other alternatives are to individualize the speed or acceleration thresholds. Individualized speed zones are based on a combination of maximal aerobic speed (MAS) which is derived from the Yo-yo intermittent recovery test level 1 or tested directly using another test, maximal sprint speed (MSS) which is derived from the maximal speed reached during training, and the anaerobic speed reserve (ASR) which corresponds to <80% of MAS, 80–100% of MAS, 100% of MAS, or 29% of ASR and ≥30% of ASR (Hunter et al., 2015; Rago et al., 2019a) (Table 1). For acceleration and deceleration zones, most studies used +3/−3 m·s^−2^ as threshold of intense/high acceleration or deceleration, respectively (Akenhead et al., 2016; Abbott et al., 2018a; Malone et al., 2019; Rago et al., 2019b). However, recent studies (Delaney et al., 2017; Rago et al., 2019b) suggest that a maximum threshold of +2/−2 m·s^−2^ should be preferred over +3/−3 m·s^−2^. Moreover, a limitation of these thresholds is that the speed is not known from which accelerations/decelerations actually begin (Rago et al., 2019a) (Table 1). Individual speed thresholds which should be adjusted according to the individual aerobic capacity show higher associations with perceptual responses to training loads compared with arbitrary speed thresholds (Rago et al., 2019a). It has to be acknowledged though that both methods showed similar sensitivity in depicting players' locomotor abilities. It seems though that the two methods should not be used interchangeably (Rago et al., 2020). The use of individualized GPS parameters (e.g., *acceleration or speed threshold*) transcribe the individual capacity of a player (Abbott et al., 2018b). A prerequisite of this approach is to regularly evaluate players' maximal aerobic speed and maximal sprint speed over the course of a season (Rago et al., 2019a). In the “real world” of professional soccer, it is not always possible to evaluate players' capacities to quantify the training load using individualized GPS parameters (Carling et al., 2018).

In summary, it can be recommended to determine relevant GPS parameters, based on arbitrary or individualized thresholds, as they will fit well with the training programs and their foundations (Rago et al., 2019b).

## Planning the Training Load for the Whole Team While Respecting Individualization

### The Importance of Analyzing Game Performance for Determining Training Load Reference Values for Each Player

The specific physical activity profile of individual players during games is used to plan monthly, weekly, and daily external TL, according to the physical demands of each player recorded during in-season games (Stevens et al., 2017; Martín-García et al., 2018). For this purpose, the game reference (G*ref*) values have to be quantified individually (Akenhead and Nassis, 2016). Individual G*ref* include every official game of the current and the previous season. For determining GPS parameters used to monitor external TL, G*ref* is arbitrarily calculated as the mean of the five best values recorded during official games (Table 2) as players are prepared for the most physical demanding games. For new players, as a rule, G*ref* is created, or according to the reference data from the literature (Ingebrigtsen et al., 2015; Suarez-Arrones et al., 2015) or with the values of the previous season on the same competition level.

**Table 2:** Game reference values from four different pitch positions.

	**Total distance (m)**	**HSR (m)**	**SPR (m)**	**Acc (n)**	**Dec (n)**	**Max speed (km/h)**
CB	9962	493	217	29	22	34
MD	12045	928	353	39	62	34
W-MD	10340	756	378	47	55	34
Attackers	10415	643	348	36	59	35

*CB, Center back; MD, Midfielder; W-MD, Wide Midfielder; HSR, high speed running; (m), meters; (n), number; Acc, number of accelerating efforts up to 3 m/s^2^; Dec, number of decelerating efforts up to−3 m/s^2^; Max speed, maximal speed*.

The example depicted in Table 2 highlights the values of physical demands (Under 23 UEFA Champion's League Team) according to the players' positions on the pitch during games.

### Scheduled Plans With Reference to Games Performance

Game reference values for each GPS parameter (Table 1) allow staff to program external TL at both collective and individual levels (Rago et al., 2019b). Collectively, external TL is calculated for each GPS parameter by a collective weighted factor (described below) of game reference values. G*ref* being specific to each player, calculation of external TL is individualized (Ingebrigtsen et al., 2015). For each player, external TL is calculated in meters or number of events according to the nature of the GPS parameter. For example, for high speed running, a distance to be covered in meters is calculated (Sæterbakken et al., 2019) whereas for acceleration and deceleration, the number of efforts (Varley et al., 2017) to be achieved is calculated.

It can be recommended to collectively and individually program the external TL by multiplying G*ref* by a weighting factor (e.g., *3.2 for weekly total distance*).

### Monthly

**Figure 1:**
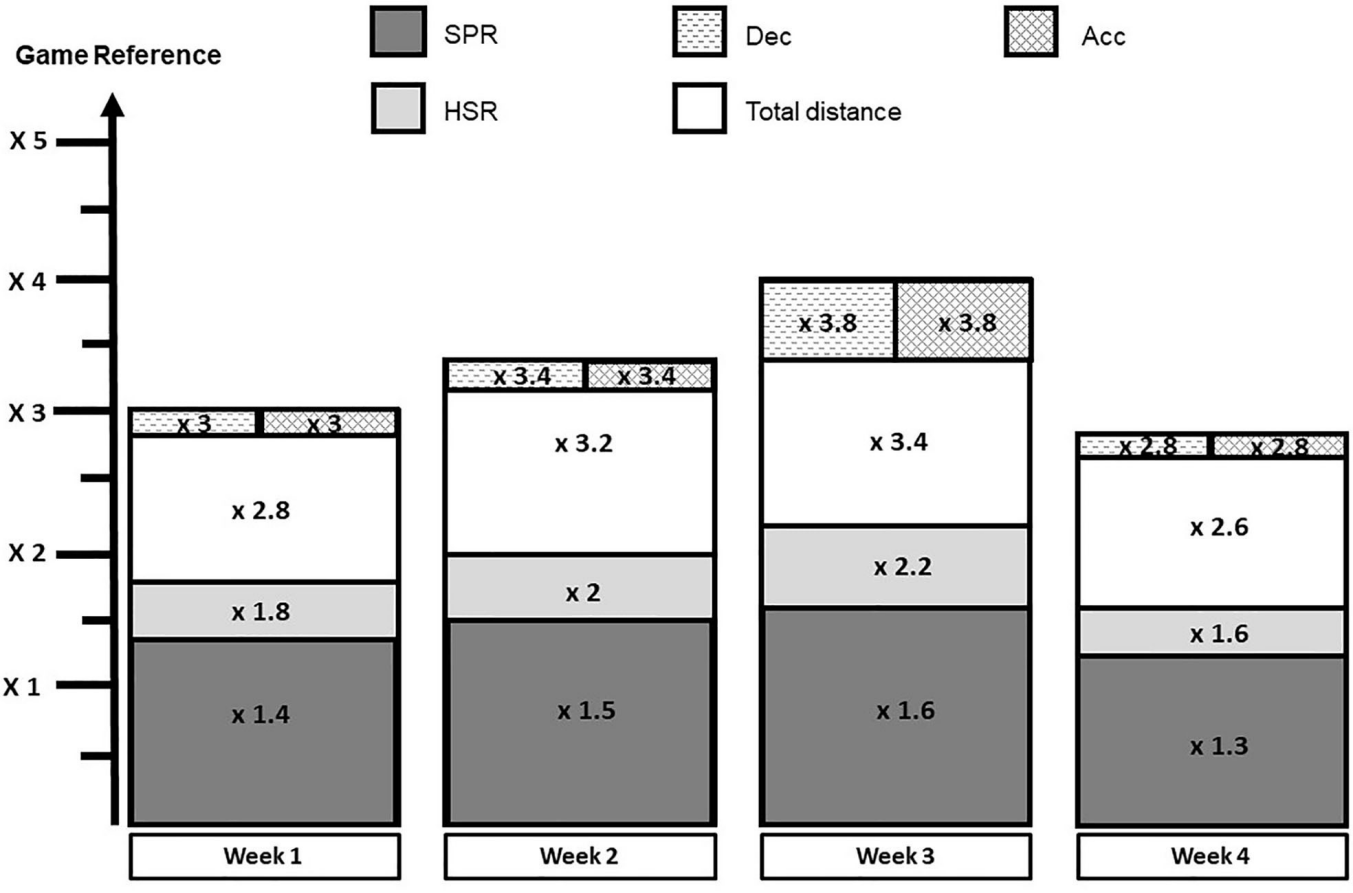
Example of a monthly collective plan. HSR, high-speed running; SPR, sprint running; Dec, deceleration; Acc, acceleration.

During the first 4 weeks, external TL increases progressively to reach the targeted high chronic (4 weeks) TL value. From data recorded in Dutch and English professional soccer leagues, chronic high total distance would be up to 111,500 m and chronic high speed running and sprint combined would be up to 3,727 and 6,173 m, respectively (Jaspers et al., 2018; Bowen et al., 2019). For bouts of acceleration and deceleration, data from the literature are difficult to use because of the filters that are applied by the different manufacturers (Varley et al., 2017). From these published data, we propose a monthly collective plan with one game per week (Figure 1). For each week and for each GPS parameter, G*ref* is weighted by a weekly factor [F*w(i)*] to calculate the weekly external TL (W*TL*) (Equation 1). F*w(i)* is arbitrarily chosen to reach a high chronic TL described in the literature (Jaspers et al., 2018; Bowen et al., 2019)

$$\begin{array}{l} {\text{W}}_{TL}=\text{F}w\left(i\right)\times \;\text{G}ref \end{array}$$

W_*TL*_ is weekly external TL; F_*w*(*i*)_ Is the weekly weighted factor; *(i)* is the number of the weeks; Gref is the game reference value.

Figure 1 illustrates a collective plan of four training weeks. Total distance, high speed running, sprint, bouts of acceleration and deceleration increase gradually during the first 3 weeks by an increase in F*w(i)* and a decrease during week 4. For example, for high speed running, F*w(i)* is 1.8, 2.0, 2.2, and 1.6 for weeks 1, 2, 3, and 4, respectively. External TL increases correspond to an increase around ~10% between weeks (Table 3). This 10% increase between weeks guarantees an ACWR between 0.80 and 1.20 (Gabbett, 2016). Examples depicted in Table 3 show the weekly data for four different pitch positions. External TL planning is a collective framework but, in the “real world,” individual particularities force to individually adapt the monthly external load (as later explained on “adjusting individually external TL”).

**Table 3:** Weekly external training load calculations for different players and GPS parameters.

	**Week 1**	**Week 2**	**Week 3**	**Week 4**	**Sum**	**Chronic TL**	**0.8 × chronic TL**	**1.5 × chronic TL**
**TOTAL DISTANCE (M)**								
CB	27892 (+8%)	31877 (+14%)	35862 (+13%)	25900 (−28%)	121532	30383	24306	45574
MD	33725 (+8%)	38543 (+14%)	43361 (+13%)	31316 (−28%)	146946	36737	29389	55105
W-MD	28953 (+8%)	33089 (+14%)	37226 (+13%)	26885 (−28%)	126153	31538	25231	47308
Attackers	29161 (+8%)	33328 (+14%)	37494 (+13%)	27079 (−28%)	127062	31765	25412	47648
**HSR (m)**								
CB	887 (+13%)	986 (+11%)	1084 (+10%)	789 (−27%)	3746	937	749	1405
MD	1671 (+13%)	1857 (+11%)	2043 (+10%)	1486 (−27%)	7057	1764	1411	2646
W-MD	1362 (+13%)	1513 (+11%)	1664 (+10%)	1210 (−27%)	5749	1437	1150	2156
Attackers	1157 (+13%)	1286 (+11%)	1414 (+10%)	1029 (−27%)	4886	1222	977	1832
**SPR (m)**								
CB	303 (+8%)	325 (+7%)	347 (+7%)	282 (−19%)	1256	314	251	471
MD	495 (+8%)	530 (+7%)	565 (+7%)	459 (−19%)	2049	512	410	769
W-MD	529 (+8%)	567 (+7%)	605 (+7%)	491 (−19%)	2192	548	438	822
Attackers	487 (+8%)	522 (+7%)	556 (+7%)	452 (−19%)	2017	504	403	756
**Acc (N)**								
CB	87 (+7%)	99 (+13%)	110 (+12%)	81 (−26%)	377	94	75	141
MD	116 (+7%)	131 (+13%)	146 (+12%)	108 (−26%)	501	125	100	188
W-MD	141 (+7%)	160 (+13%)	179 (+12%)	132 (−26%)	611	153	122	229
Attackers	107 (+7%)	121 (+13%)	135 (+12%)	99 (−26%)	462	115	92	173
**Dec (N)**								
CB	66 (+7%)	75 (+13%)	84 (+12%)	62 (−26%)	286	72	57	107
MD	185 (+7%)	210 (+13%)	235 (+12%)	173 (−26%)	803	201	161	301
W-MD	166 (+7%)	188 (+13%)	211 (+12%)	155 (−26%)	721	180	144	270
Attackers	175 (+7%)	199 (+13%)	222 (+12%)	164 (−26%)	761	190	152	285

*CB, Center back; MD, Midfielder; W-MD, Wide Midfielder; TL, training load; HSR, high speed running; (m), meters; (N), number; Acc, acceleration; Dec, deceleration*.

To summarize, external TL should be planned to progressively reach a greater, elevated chronic external TL on a monthly basis. Game reference is weighted by a weekly factor (*for example: Total distance x 2.8 on week 1, x 3.2 on week 2, x 3.4 on week 3 and x 2.6 on week 4*).

#### Weekly

The weekly training schedule is organized around the game day (GD) (Akenhead et al., 2016). Training days are scheduled based on the number of available days for training before and after the game day (i.e., minus or plus the game day; Clemente et al., 2019a). In order to adapt the external TL depending on the game physical demand (see below), the week should start at game day and finish at game day−1. Following published data (Malone et al., 2015; Akenhead et al., 2016; Stevens et al., 2017; Martín-García et al., 2018), weekly external TL is arbitrarily distributed during the days of the week (D*TL*). For each day and for each GPS parameter, G*ref* is weighted by daily arbitrary factor [F*GD(i)*] to calculate daily external TL (Equation 2).

$$\begin{array}{l} {\text{D}}_{TL}={\text{F}}_{GD\left(i\right)}\times \;{\text{G}}_{ref} \end{array}$$

D_*TL*_ is daily training load; F_*GD*(*i*)_ is daily weighting factor; *(i)* is the number of the day; G_*ref*_ is the game reference values.

**Figure 2:**
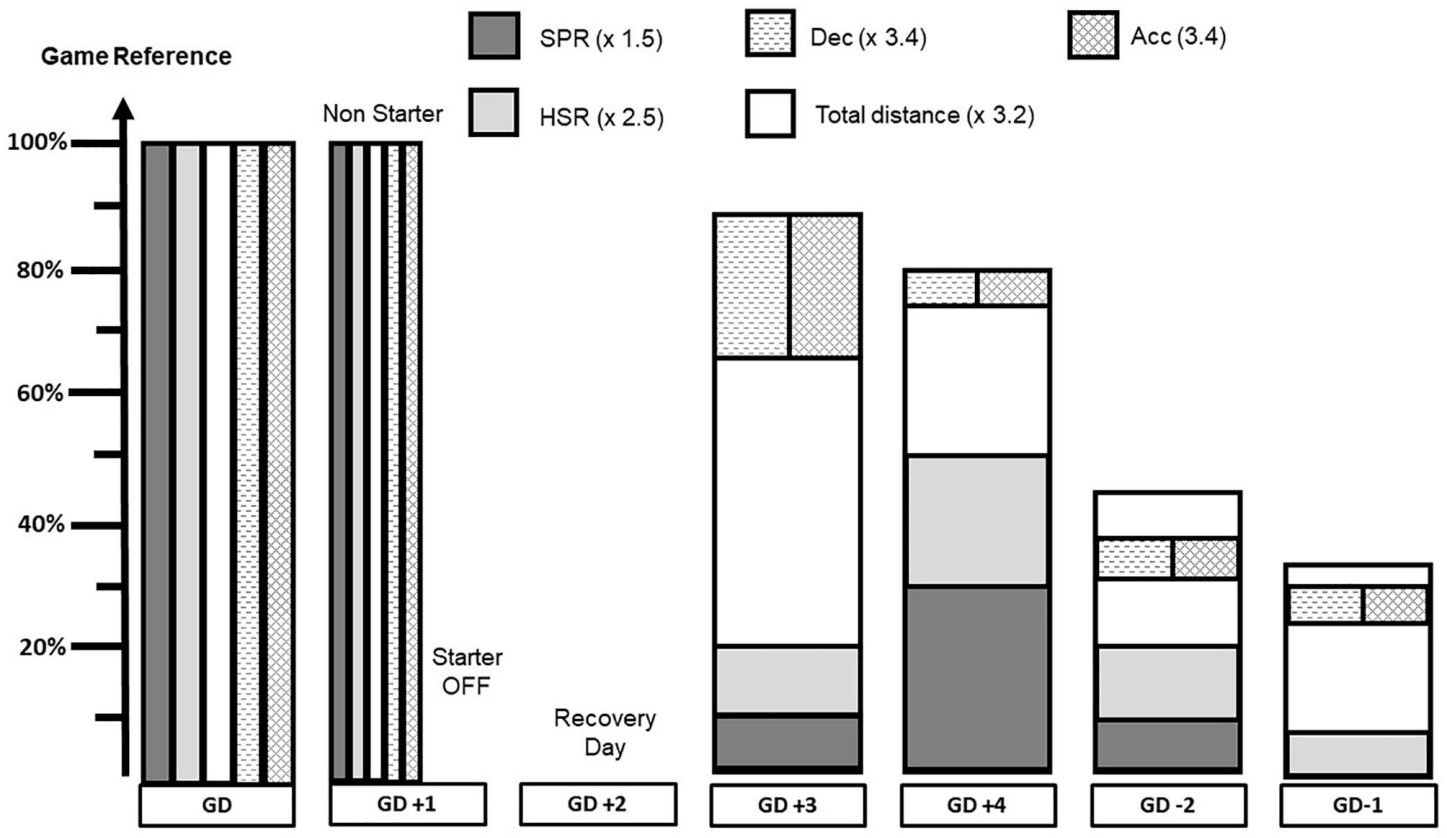
Example of a weekly collective plan (week 2) from individual match reference values. HSR, high-speed running; SPR, sprint running; Dec, decelerations; Acc, accelerations; GD, game day.

Figure 2 illustrates week 2 of a monthly collective plan. Game day +1 is devoted to recover from matches, specifically those players who played ≥60 min, while players who played ≤ 30 min perform a compensatory training session. This figure presents published data from teams in the Netherlands and Portugal (Clemente et al., 2019a), game day +3 and game day +4 are the most important days in terms of external TL whereas an important reduction in external TL is programed for game day −1.

**Figure 3:**
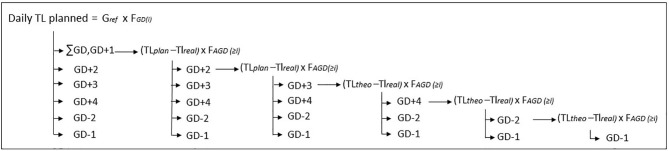
Proportional distribution of the difference between TL*plan* and TL*real* for each day (see text of the paper for abbreviation explanation).

As for monthly external TL planning, the weekly external TL planning is a collective framework but, in the “real world,” for each day and GPS parameter, a difference between the planned (TL*plan*) and the realized (TL*real*) TL may occur and is referred to as TL*diff(i)* (Equation 3). For each day of the week, the difference between TL*plan* and TL*real* is distributed proportionally at F*GD(i)* on the subsequent days of the week (Figure 3). A weighted factor entitled F*AGD(i)* is also calculated (Equation 4). Finally, for each day, TL*adj(i)* is calculated and distributed proportionally on the subsequent days of the week (Equation 5).

$$\begin{array}{l} {\text{TL}}_{diff\left(i\right)}=\left({\text{TL}}_{plan-}\;{\text{TL}}_{real}\right) \end{array}$$

TL*adj(i)* is the external TL difference between TL*plan* and TL*real* on a day *(i)*

$$\begin{array}{l} \text{F}AGD\left(i\right)=\text{F}GD\left(i\right)/\;\sum \left(\text{F}GD\geq \left(i\right)\right) \end{array}$$

Whereas; F*AGD(i)* is the weighted factor for the difference between daily TL*plan* and daily TL*real*; F*GD(i)* is the daily weighted factor; ∑(F*GD*≥*(i)*) is the sum of the daily weighted factor on the subsequent days of the week; *(i)* is the number of the day; ≥*(i)* is posterior and include *(i)*.

$$\begin{array}{l} \text{TL}adj\left(i\right)=\text{TL}diff\left(i\right)\times \;\text{F}AGD\left(i\right) \end{array}$$

Table 4 shows, for 4 different pitch positions, an example of proportional distribution calculations for total distance, high speed running, sprint, acceleration and deceleration after game day. On game day, TL*plan* is G*ref* whereas TL*real* is random. On this table, in brackets, the individual distribution of match external TL during the week is expressed as percentages.

**Table 4:** Example of weekly external TL distribution adjusted in terms of game physical demands, for each GPS parameters.

	**W*TL***	**GD**	**GD +3**	**GD +4**	**GD-2**	**GD-1**
	**Total distance (x3.2)**	**Total distance**	**Total distance (x0.65)**	**Total distance (x0.75)**	**Total distance (x0.45)**	**Total distance (0.35)**
CB	*31877*	*9832 (*–*1%)*	*6513 (+0.3%)*	*7516 (+0.34%)*	*4509 (+0.2%)*	*3507 (+0.16%)*
MD	*38543*	*11045 (*–*8%)*	*8125 (+2.4%)*	*9375 (+2.6%)*	*5625 (+1.6%)*	*4375 (+1.4%)*
W-MD	*33089*	*9998 (*–*3%)*	*6823 (+0.89%)*	*7872 (+1.02%)*	*4723 (+0.61%)*	*3574 (+0.48%)*
Attackers	*33328*	*10254 (*–*2%)*	*6816 (+0.59%)*	*7866 (+0.68%)*	*4720 (+0.41%)*	*3671 (0.32%)*
	**HSR (x2)**	**HSR**	**HSR (x0.2)**	**HSR (x0.5)**	**HSR (x0.2)**	**HSR (x0.1)**
CB	*985*	*296 (*–*40%)*	*138 (+8%)*	*345 (+20%)*	*138 (+8%)*	*69 (+4%)*
MD	*1857*	*743 (*–*20%)*	*222 (+4%)*	*557 (+10%)*	*223 (+4%)*	*111 (+2%)*
W-MD	*1513*	*908 (+20%)*	*121 (*–*4%)*	*303 (*–*10%)*	*121 (*–*4%)*	*61 (*–*2%)*
Attackers	*1286*	*643 (0%)*	*129 (0%)*	*322 (0%)*	*129 (0%)*	*64 (0%)*
	**SPR (x1.5)**	**SPR**	**SPR (x0.1)**	**SPR (x0.3)**	**SPR (x0.1)**	**SPR**
CB	*325*	*130 (*–*40%)*	*39 (+8%)*	*117 (+24%)*	*39 (+4%)*	–
MD	*530*	*283 (*–*20%)*	*49 (+4%)*	*148 (+12%)*	*49 (+4%)*	–
W-MD	*567*	*454 (+20%)*	*23 (*–*4%)*	*68 (*–*12%)*	*23 (-4%)*	–
Attackers	*522*	*348 (0%)*	*35 (0%)*	*104 (0%)*	*35 (0%)*	–
	**Acc (x3.4)**	***Acc***	**Acc (x0.9)**	**Acc (x0.8)**	**Acc (x0.4)**	**Acc (x0.3)**
CB	*99*	*32 (+10%)*	*25(*–*3.75%)*	*22 (*–*3.33%)*	*11 (*–*1.67%)*	*8 (*–*1.25%)*
MD	*131*	*36 (*–*6%)*	*36 (+2.25%)*	*32 (+2%)*	*16 (+1%)*	*12 (+0.75%)*
W-MD	*160*	*46 (*–*2%)*	*43 (+0.75%)*	*38 (+0.67%)*	*19 (+0.33%)*	*14 (+0.25%)*
Attackers	*121*	*34 (*–*4%)*	*33 (+1.5%)*	*29 (+1.33%)*	*14 (+0.67%)*	*11 (+0.5%)*
	**Dec (x3.4)**	**Dec**	**Dec (x0.9)**	**Dec (x0.8)**	**Dec (x0.4)**	**Dec (x0.3)**
CB	*75*	*25 (+14%)*	*19 (*–*5.25%)*	*17 (*–*4.67%)*	*8 (*–*2.33%)*	*6 (*–*1.75%)*
MD	*210*	*58 (-6%)*	*57 (+2.25%)*	*51 (+2%)*	*25 (+1%)*	*19 (+0.75)*
W-MD	*188*	*48 (-13%)*	*53 (+4.9%)*	*47 (+4.33%)*	*23 (+2.17%)*	*18 (1.6%)*
Attackers	*199*	*54 (-8%)*	*53 (+3%)*	*48 (+2.7%)*	*24 (+1.3%)*	*18 (+1%)*

*CB, Center back; MD, Midfielder; W-MD, Wide Midfielder; HSR, high-speed running expressed in meters; SPR, Sprint running expressed in meters; WTL, weekly training load; GD, game day. See abbreviation list at the beginning of the paper for definition of abbreviations used and not denoted here*.

On game day −1, at the end of the week, TL*plan* is the result of the recorded TL*real* on the previous day. It serves as an information for the technical staff to adapt, if necessary, the last collective training session. For example, if at game day−1, high speed running distance recorded [game day + (game day +2) + (game day +3) + (gamed day +4) + game day−2)] is collectively higher than what was planned, the technical staff can choose to decrease the pitch area to diminish high speed running distance while preserving the tactical objective.

To summarize, we recommend the external TL to be planned on a weekly basis. Game reference has to be weighted using a daily factor (e.g., *0.6*). This should be realized on an individual level and adjusted according to an algorithm that takes TL*plan* and TL*real* into account.

#### Daily

**Figure 4:**
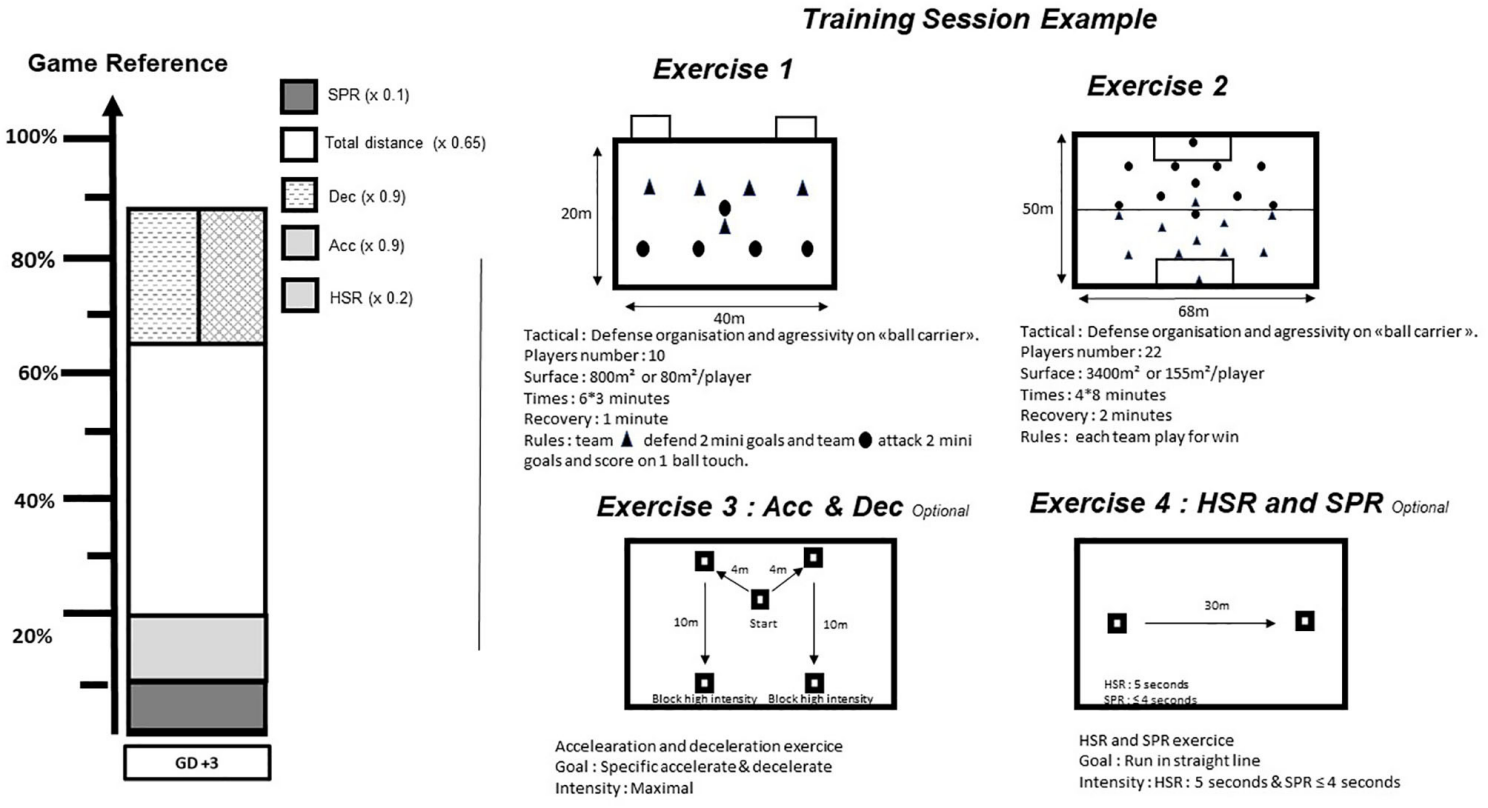
Example of a daily team training session. HSR, high- speed running; SPR, sprint running; Dec, decelerations; Acc, accelerations; GD, Game day.

Before training, in accordance with the technical staff, we program the training session to reach the collective objective of the day with respect to the planned weekly external TL. Continuous feedback during the training session allows to adjust in “real time,” both collectively and individually, the external TL in relation to TL*plan*. This adjustment could be met collectively by modifying a game rule, the pitch area, and the duration of exercise; and individually by adding a specific physical exercise in order to reach TL*plan* of the day. Collective exercises and specific physical exercises are programmed to meet the physical demands of the match. Thereby, for each player, the distance covered for each high-speed running, sprint, acceleration and deceleration in m/min should be continuously assessed to verify the specificity of an exercise. Figure 4 shows an example of a training session in game day +3 of the second week of the monthly planned external TL. G*ref* of total distance, high speed running, sprint, bouts of acceleration and deceleration are weighted by 0.65, 0.2, 0.1, 0.9, and 0.9, respectively. In other words, total distance, high speed running, sprint, acceleration and deceleration represent 65, 20, 10, 90, and 90 percent of G*ref*, respectively. This training session combined physical (i.e., acceleration/deceleration specific endurance), technical (i.e., play with high pressure), tactical (i.e., press zone and organization formation), and mental (i.e., concentration) goals which were defined by the technical staff. At the end of the training session, two optional specific physical exercises were assigned to the players who did not reach TL*plan*. For this example, the values for the four different positions are presented in Table 5.

**Table 5:** Individual external training load value.

	**GD** **+3 TL*****plan***				
	**TD (m)**	**HSR (m)**	**SPR (m)**	**Acc (n)**	**Dec (n)**
CB	6513	138	39	25	19
MD	8125	223	49	36	57
W-MD	6822	121	23	43	53
Attackers	6817	141	41	33	54

*CB, Center back; MD, Midfielder; W-MD, Wide Midfielder; HSR, high-speed running; SPR, Sprint running; TD, Total distance; Acc, accelerations; Dec, Decelerations; m, meters; n, numbers; TLplan, training load planned; GD, match day*.

Hence, it can be recommended to build the training sessions to reach the TL*plan*. Before the training session starts, the targeted TL should be calculated for each player individually. In addition, specific exercises could be prescribed which help the player to reach the calculated TL.

#### Adjusting Individually External TL

The great heterogeneity of the team in terms of age, physical conditioning, history of injry, etc., makes it necessary to individualize external TL for each player. First, external TL is programmed by the collective weekly weighted factor [F_*w*(*i*)_] and the daily weighted factor [F_*GD*(*i*)_]. Subsequently, in order to meet the needs of each player, F_*w*(*i*)_ and F_*GD*(*i*)_ should be individualized, either to increase or to reduce the external TL with respect to the collective external TL. Physical performance and training load adaptation assessments are used to adjust individually the external TL, on a monthly, weekly, and daily basis. Monthly, a submaximal aerobic test (e.g., 4 min at 12 km/h) and countermovement jumps provide information about the aerobic and neuromuscular performance status of the players (Halson, 2014; Buchheit et al., in press). Weekly at game day +3, heart rate variability during an orthostatic stand test (Ravé and Fortrat, 2016; Ravé et al., 2020), and blood creatine kinase (Hader et al., 2019) provide information about recovery from games. Daily, before the training a wellbeing questionnaire (e.g., muscular damage, fatigue, sleep quality) (Malone et al., 2018b) and after the training, the session rating of perceived exertion [e.g., CR-10 Borg scale; (Malone et al., 2018b)] provide information on how each player perceives strain and adaptation of each training session. All these information help practitioners to make the “right decision” about the management of external TL affecting the player(s). This prescription is dynamic, adaptable and it can be updated and adjusted daily according to the “real world” if unique situations occur.

To summarize, coaches can individually adjust the weighted factor depending on the results of physical performance tests together with the observed external TL adaptations. The continuous collection of internal and external TL data on a monthly, weekly, and daily level including data from the soccer game will help to better manage (e.g., increase or decrease) TL.

#### Limitations, Strengths, and Practical Applications

This practical approach tries to build a theoretical framework using knowledge from sport science to monitor training load during “real world” soccer practice. However, some limitations must be acknowledged concerning the weighted factors discussed [e.g., F*w(i)* and F*GD(i)*]. More specifically, these factors were chosen, for the team, arbitrarily from published data and from existing monitored data. Future studies are needed to more precisely define and validate the scientific process to access these weighted factors [e.g., F*w(i)* and F*GD(i)*] for accuracy. However, the practical approach discussed herein is, to our knowledge, the first publication attempting to combine the scientific recommendations and actual coaching experience on the field. This combined approach can open up working perspectives for practitioners for external TL prescription. In practice, it is important to be sure of the reliability of the used GPS data. Furthermore, players should keep the same device all season during both training sessions and games. After choosing the relevant GPS parameters, both arbitrarily and with individualized thresholds, the parameters used should be the same over the whole season and it is not recommended to change the parameters. The game reference for each GPS parameter used is specific for each player. The collective plan of external TL is updated on a monthly, weekly, and daily bases. Finally, the adjustment of the external TL of a player depends on the difference between TL*plan* and TL*real* while considering the results from physical performance tests and training load adaptations.

## Conclusions

GPS is a valid, reliable and relevant tool for tracking the external TL in professional soccer. Previous scientific recommendations have highlighted the importance of monitoring the external TL to reduce injury risk and optimize players' physical performance. In this opinion paper, we have proposed an approach on how to use GPS data to analyze, prescribe, and control the external TL in elite soccer, both collectively (i.e., team) and individually.

## Author Contributions

GR and HZ conceived and designed the idea. All authors wrote and revised the manuscript. All authors read and approved the manuscript.

## Conflict of Interest

GR is employed by company Toulouse Football Club (Toulouse, France). The remaining authors declare that the research was conducted in the absence of any commercial or financial relationships that could be construed as a potential conflict of interest.

## Abbreviations

Acc: number of acceleration
ACWR: acute chronic workload ratio
Dec: number of deceleration
D*TL*: daily external training load
F*AGD(i)*: weighting factor of the difference between the external TL planned and external TL realized
F*GD(i)*: daily weighting factor
F*w(i)*: weekly weighting factor
GD: Game Day
GPS: Global Positioning Systems
G*ref*: game reference
HSR: high speed running
M/min: meter per minute
Max speed: maximal speed
SPR: sprint running
TL: training Load
TL*adj(i)*: the external TL difference between the external TL planned and external TL realized
TL*plan*: the external TL planned
TL*real*: external TL realized
VO_2_max: maximal oxygen consumption
W*TL*: weekly external training load.
